# G1 Dynamics at the Crossroads of Pluripotency and Cancer

**DOI:** 10.3390/cancers15184559

**Published:** 2023-09-14

**Authors:** Dalia Fleifel, Jeanette Gowen Cook

**Affiliations:** Department of Biochemistry & Biophysics, The University of North Carolina at Chapel Hill, Chapel Hill, NC 27599, USA; dalia.fleifel@unc.edu

**Keywords:** cell cycle, cyclin-dependent kinase (CDK), origin licensing, genome instability, pluripotent stem cells (PSCs), minichromosome maintenance (MCM), DNA replication

## Abstract

**Simple Summary:**

The cell division cycle is tightly regulated to ensure faithful and complete DNA replication. A critical cell cycle phase is G1 in which cells prepare DNA for replication in S phase. Interestingly, stem cells and cancer cells have both similarities and differences in their cell cycle regulatory mechanisms. In this review, we address the role of various cell cycle regulators in controlling the dynamics of G1 phase in stem cells and cancer cells. We also discuss recent advances in understanding how core pluripotency factors regulate the cell cycle and play dual roles in stem cell pluripotency and in cancers where they are aberrantly expressed. A better understanding of these common regulatory networks could offer potential new therapeutic avenues for cancer.

**Abstract:**

G1 cell cycle phase dynamics are regulated by intricate networks involving cyclins, cyclin-dependent kinases (CDKs), and CDK inhibitors, which control G1 progression and ensure proper cell cycle transitions. Moreover, adequate origin licensing in G1 phase, the first committed step of DNA replication in the subsequent S phase, is essential to maintain genome integrity. In this review, we highlight the intriguing parallels and disparities in G1 dynamics between stem cells and cancer cells, focusing on their regulatory mechanisms and functional outcomes. Notably, SOX2, OCT4, KLF4, and the pluripotency reprogramming facilitator c-MYC, known for their role in establishing and maintaining stem cell pluripotency, are also aberrantly expressed in certain cancer cells. In this review, we discuss recent advances in understanding the regulatory role of these pluripotency factors in G1 dynamics in the context of stem cells and cancer cells, which may offer new insights into the interconnections between pluripotency and tumorigenesis.

## 1. Introduction

In the complex world of cellular processes, two seemingly distinct entities—stem cells and cancer cells—converge at a surprising crossroad: their cell cycle dynamics. Stem cells during embryonic development are characterized by rapid proliferation which is reminiscent of the excessive and uncontrolled proliferation that is a hallmark of cancer [1,2]. On the flip side, adult stem cells reside in our organs in a dormant, non-proliferating state, which upon injury, can switch into a proliferative state to maintain tissue homeostasis [3]. Similarly, specific cancer cells reside in a slow cycling or dormant state within tumors, express stemness markers, and confer resistance to anti-proliferative cancer drugs [4]. However, while these commonalities exist, we highlight in this review both distinct and similar cell cycle and DNA replication pathways employed by stem cells versus cancer cells, which could shape their divergent destinies.

The canonical mammalian cell division cycle is divided into four sequential phases: G1, S, G2, and M. Accurate transition from G1 into S phase controls cell proliferation and ensures faithful DNA replication in S phase, defects in which cause genome instability. Cell cycle regulators, such as periodically expressed cyclins, the cyclin-dependent kinases to which they bind and activate (CDKs), and CDK inhibitor proteins, govern cell cycle progression in an orderly and timely manner (see also Section 3 below). In this review, we highlight the similarities and differences between stem cells and cancer cells in the expression levels and activities of G1 cyclin-CDK complexes, and their protein inhibitors. Additionally, we discuss the dynamics of DNA replication origin licensing, one of the rate-limiting steps in cell proliferation, shedding light on the intriguing concept of dormant origins in both cell types.

A significant portion of this review centers around pluripotency factors, such as SOX2, OCT4, KLF4, and the pluripotency reprogramming facilitator c-MYC. These factors are well-known for their role in governing the pluripotency network in stem cells, and intriguingly, they are also aberrantly expressed in specific cancer cells (reviewed in [5,6,7,8]). We examine how these pluripotency factors are central to the common G1 regulation in stem cells and some cancer types. A comprehensive understanding of these complex processes holds great promise for identifying common pathways between tumorigenesis and stem cell pluripotency, potentially leading to novel therapeutic strategies and translational advancements.

## 2. G1 Dynamics in Stem Cells and Cancer

### 2.1. Balancing G1 Phase Length and Origin Licensing Dynamics

**Figure 1:**
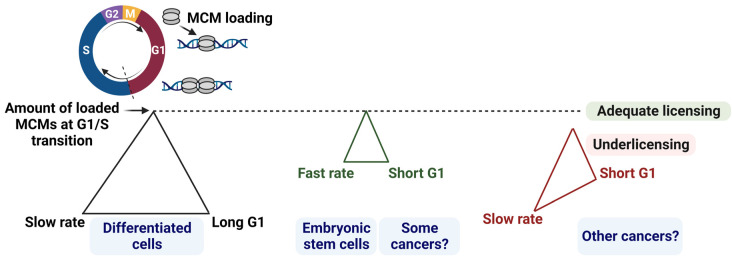
G1 length, MCM loading amount, and rates must be tightly coupled to maintain genome integrity. The figure illustrates the conceptual relationships among the three parameters. MCM loading increases gradually in G1 until it reaches the maximum loaded amount of MCMs at the G1/S transition (depicted with the dotted line). (Left triangle): G1 length and MCM loading rates must be tightly coordinated to achieve adequate origin licensing. (Middle triangle): a short G1 must be counterbalanced with fast MCM loading rates to achieve adequate licensing at the G1/S transition, and fast licensing is a characteristic of embryonic stem cells. The rates of licensing in at least a subset of cancer cells are expected to be fast although they have not yet been directly measured in different cancer types. (Right triangle): if MCM loading rates are not fast enough in cells with a short G1, cells will suffer underlicensing and incomplete DNA replication [10]. Created with BioRender.com, accessed on 29 August 2023.

An essential molecular event in G1 phase, known as origin licensing, requires that many thousands of minichromosome maintenance (MCM) complexes are loaded onto mammalian DNA to “license” multiple sites for DNA replication. Activation or “firing” of licensed origins occurs only in S phase and involves the conversion of loaded MCM complexes into active DNA helicases to initiate DNA replication. Origin licensing is strictly prohibited in S phase to prevent a form of self-inflicted DNA damage known as “re-replication”; thus, G1 phase is the only window available for origin licensing. Interestingly, G1 lengths vary greatly across multiple cell types. As a consequence of origin licensing restriction to G1, a short G1 needs to be counterbalanced by rapid licensing rates to ensure there are enough loaded MCMs to complete DNA replication. Otherwise, cells with a short G1 and slow licensing rates are prone to insufficient licensing or “under licensing”, which sensitizes cells to DNA replication stress and eventually leads to genome instability (reviewed in [9]) as illustrated in Figure 1. Although the licensing mechanism has been extensively studied, how licensing rates are controlled, especially in cells with a short G1, is still unclear.

### 2.2. Consequences of a Short G1: A Common Feature in Stem Cells and Some Cancers

At embryonic stages, the mammalian cell cycle is remarkably rapid with extremely short gap (G1 and G2) phases [11]. Pluripotent stem cells (PSCs) respond to differentiation cues during the G1 phase; thus, a short G1 phase and rapid proliferation protect pluripotency by reducing the time window available to respond to differentiation signals [12]. Cells committed to differentiation typically have long G1 phases [13] and slow rates of origin licensing [10]. Conversely, somatic cells that undergo successful reprogramming into induced pluripotent stem cells (iPSCs) via the ectopic expression of the four “Yamanaka factors” (OCT4, SOX2, KLF4, and c-MYC), acquire a short G1 and shift to rapid proliferation early during the reprogramming process [14,15,16]. This observation hints that rapid cycling enhances the efficiency of reprogramming. Moreover, we have previously discovered that PSCs rapidly license origins to counterbalance their short G1 phase and achieve high MCM loading at the start of S phase [10]. Mechanistic insights into how origin licensing rates and G1 length are coupled with developmental signals to establish or maintain pluripotency are still lacking, however. 

In human cancers, a short G1 phase is a typical outcome of oncogene amplification such as *CCNE1* (the gene encoding cyclin E1) and *AML-1* [17,18]. Oncogene-induced premature S phase entry leads to aberrant origin firing in intragenic regions, replication–transcription collisions, and depletion of nucleotide pools, which result in replication fork collapse and DNA replication stress [19,20,21]. On the other hand, the G1 length in normal cell cycles is long enough for the transcription machinery to inactivate intragenic origins before DNA replication starts in S phase. Acute cyclin E1 overproduction also contributes to replication stress by inducing underlicensing. However, cells can adapt to the sustained high levels of cyclin E1, partially restore normal levels of licensing, and continue proliferation [22]. Whether other oncogenes can shorten G1 but also induce rapid origin licensing would be interesting to explore. 

Although a short G1 is a common characteristic between cancer cells and PSCs, PSCs must specifically employ robust mechanisms to counteract replication stress in S phase and safeguard their genome integrity against any mutations, which is indispensable for proper embryonic development. ESCs utilize unique protein regulatory mechanisms and cellular pathways to restart stalled forks [23], accurately repair DNA damage [24,25], and stimulate apoptosis when needed [26]. Moreover, ESCs load an excessive amount of MCMs in G1 phase as a back-up for the stalled and collapsed forks in S phase [27]. However, ESCs have elevated levels of the transcription factor MYBL2 (alias, B-MYB), which functions with the cell cycle checkpoint kinase, ATM, to prevent excessive origin firing and genome instability [28]. These high-fidelity rescue mechanisms might be lacking or altered in cancer cells to suppress DNA damage checkpoints and tolerate replication stress [29]. In summary, although a short G1 in both PSCs and some cancer cells drives rapid cellular proliferation, the short G1 in cancer cells is a consequence of genetic mutations such as oncogene activation, whereas it is a normal feature that maintains stem cell pluripotency.

### 2.3. Cancer Resistance: Quiescence or a Cycling Stem Cell-like State?

On the opposite side of rapid proliferation, quiescence (or G0), is a reversible state where cells exit G1 phase and enter proliferation arrest. However, these cells retain their ability to re-enter the cell cycle in response to cues such as mitogen stimulation or injury [30]. In contrast to rapidly proliferating embryonic stem cells, adult stem cells reside in different mammalian tissues in a quiescent state. When stimulated, adult stem cells can transition into a proliferative state, renew themselves, and generate either quiescent or actively dividing cells to facilitate tissue maintenance, repair, and regeneration [3].

Similarly, cancer cell dormancy is a process in which cancer cells enter a pathological state of quiescence. These dormant cells tolerate anti-proliferative cancer drugs and can exit a long G0 phase into the cell cycle to re-initiate tumors [4]. In quiescence, the DYRK1A protein kinase is activated, DYRK1A phosphorylates cyclin D (promotes proliferation) and p27 (inhibits proliferation)—among other targets—degrading the former and stabilizing the latter, which contributes to cancer dormancy [31,32]. Deleting DYRK1A in ovarian cancer results in the loss of viability of dormant cells in tumor spheroids [33]. The field is still trying to define the underlying mechanisms that trigger the G0 exit of these dormant cells. 

It is important to distinguish between dormant cancer cells and cancer stem cells because they are often mistakenly used interchangeably [4]. First, a dormant cancer cell is a reversibly arrested cell, whereas a cancer stem cell is a slowly cycling cell [34]. Second, dormant cancer cells are differentiated, whereas cancer stem cells can self-renew: a characteristic of stem cells [35]. Third, cancer stem cells, but not all dormant cancer cells, express stemness markers such as OCT4 and SOX2. Similar to dormant cancer cells, cancer stem cells are also resistant to anti-cancer drugs and promote tumor relapse in patients [4]. However, the drug resistance mechanisms might be different between the two categories.

**Figure 2:**
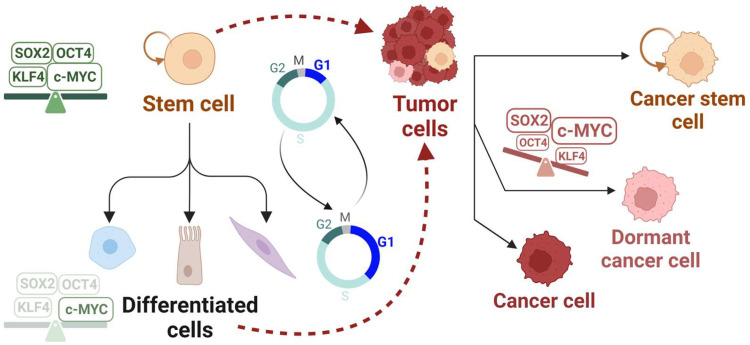
Pluripotency factors’ network could account for similarities between stem and cancer cell cycle dynamics. A stem cell is characterized by its ability to self-renew (depicted by the circular arrow) and differentiate into cells of multiple lineages. Stem cells or differentiated cells (unless terminally differentiated) can acquire genetic or epigenetic alterations, among several other factors, that could result in transformation (depicted by the dashed red arrows) [2]. An intricate balance of pluripotency factors (SOX2, OCT4, and KLF4, among others) together with c-MYC in stem cells, versus their aberrant unbalanced expression in rare tumor populations, could contribute to the short G1 and altered cell cycle dynamics through poorly defined mechanisms. (Right side): Some tumor types comprise a heterogenous mixture of cells; (Top) cancer stem cell: can self-renew (depicted by the circular arrow), cycle slowly and contribute to tumor resistance; (Middle) dormant cancer cell: quiescent, can initiate tumors and contribute to tumor resistance; (Bottom) actively dividing cancer cell [4]. A short G1 phase is a common characteristic among stem cells and some cancers, while a long G1 phase is typical of normal differentiated cells [10]. Created with BioRender.com, accessed on 29 August 2023.

Do adult stem cells and dormant cancer cells share stemness features that render them capable of re-entering the cell cycle and repopulating tissues (Figure 2)? Can we predict when and how they will re-enter the cell cycle based on specific expression patterns of cell cycle or stemness markers? How do the cycling cancer stem cells resist drugs that target the cell cycle machinery? Answering these questions will be invaluable to better understand tissue repair, tumor relapse, and drug resistance.

## 3. Cell Cycle Regulators of G1 Progression in Stem Cells and Cancer

The cell division cycle is a series of unidirectional, tightly-regulated events that lead cells through a growth phase (G1), the DNA synthesis phase (S), a second growth phase (G2) that directly precedes chromosome segregation, and the cell division phase, or mitosis (M). 

Core proteins known as cyclins, cyclin-dependent kinases (CDKs), and cyclin-dependent kinase inhibitors (CDKIs) govern cell cycle progression in somatic cells by exhibiting an oscillatory activity. When cyclin D levels are elevated in early G1, cyclin D binds and activates CDK4/6 which then phosphorylates Rb—a member of the pocket protein family of transcriptional inhibitors. The E2F1-3a transcription factors control the genes required for S phase, and these genes are repressed by Rb bound to E2F. Rb phosphorylation results in de-repression of the E2F-regulated genes, one of which is *CCNE1*. Cyclin E protein then binds and activates CDK2 to further phosphorylate Rb. The role of CDK4/6 in G1 progression through to the S phase has been recently re-examined. The prevailing model has been initial Rb phosphorylation mediated by CDK4/6, followed by Rb hyperphosphorylation by CDK2 in G1. High CDK2 activity initiates a CDK2-Rb positive feedback that drives cell cycle progression independently of CDK4/6 activity [36]. However, recent studies indicate that persistent CDK4/6 activity can be required to maintain both Rb hyperphosphorylation throughout all of G1 phase [37,38], and CDK2 activity in S and G2 phases [39]. Different models for the regulation of the CDK-Rb-E2F pathway and G1/S progression are presented in this excellent review [40]. 

In S phase, cyclin A levels are elevated, where it binds to CDK2 to regulate the progression through S phase; cyclin A is also the product of an E2F-regulated gene. In G2/M, cyclin A-CDK1 and cyclin B-Cdk1 activities control the progression into mitosis. Thus, each CDK protein kinase activity is highly dependent on the expression level and timing of its cognate cyclin partner in different cell cycle phases (reviewed in [41]).

Moreover, CDK inhibitor proteins can bind to their respective CDKs to inhibit their association with cyclins or block access to substrates, and prevent the phosphorylation of specific targets in different cell cycle phases. There are two classes of CDK inhibitors: (1) inhibitors of CDK4 (INK4), which also inhibit CDK6—INK4s include p15, p16, p18, and p19—and (2) kinase inhibitor proteins (KIPs), which inhibit cyclin-CDK complexes. KIPs include p21, p27, and p57 (reviewed in [42,43]).

It is important to note that both the cyclins and CDK inhibitor proteins are themselves cell-cycle-regulated, i.e., their expression levels oscillate during cell cycle progression. Thus, experimentally observed differences in their expression levels, measured using assays such as immunoblotting or transcriptomics in two different asynchronous populations with different cell cycle distributions, might only be a symptom, not a cause of cell cycle changes. For example, cyclin A levels increase during S phase and decrease in late G2/M. Thus, a population of cells that has predominantly S phase cells (such as PSCs) will generate a higher overall cyclin A signal compared to another population that has predominantly G1 phase cells (such as differentiated cells). In Section 3, we will discuss the similarities and differences between stem cells and cancer cells with regard to G1 cyclins, CDKs, and CDKIs regulation.

### 3.1. Cyclins and CDKs

As discussed earlier, pluripotent stem cells and some cancer cells cycle rapidly with very short gap (G1 and G2) phases. Interestingly, the late G1 cyclin, cyclin E, is constitutively highly expressed in murine ESCs (mESCs); however, it switches back to its oscillatory nature upon differentiation [44]. The levels of the ubiquitin ligase substrate adapter, FBXW7, which targets cyclin E for degradation, are intrinsically low, whereas transcription factors, which are key regulators of pluripotency such as c-MYC, TFCP2L1, KLF4, and ESRRB, positively regulate cyclin E expression in mESCs [45,46]. Consequently, CDK2 is constantly active, Rb is hyperphosphorylated, and E2F activity is constitutively high, which could explain the truncated G1 phase in mESCs (<3 h) [41]. 

A short G1, through constitutive high cyclin expression and CDK activity, would help maintain pluripotency and block differentiation by limiting the time available to respond to differentiation cues in G1 [12,47]. Direct evidence for this premise came from the genetic ablation of all G1 cyclins (cyclins D and E) in mESCs, which led to the loss of pluripotency and trophectoderm differentiation. Moreover, the G1 cyclin-CDK complexes together phosphorylate key pluripotency factors and prevent their proteasomal degradation [48]. For example, CDK2 phosphorylates SOX2 (at Serines 39 and 253), which is necessary for establishing pluripotency during reprogramming into iPSCs, but is dispensable for the self-renewal and cell cycle progression of ESCs [49]. Moreover, CDK2 depletion in hESCs results in differentiation towards extra-embryonic lineages due to a decreased expression of pluripotent transcription factors (OCT4, SOX2, NANOG) or markers (SSEA-4) [50]. In addition, small molecule inhibitors of CDK2 and CDK4/6 diminish reprogramming efficiency [51]. Thus, high CDK activity promotes pluripotency by directly regulating pluripotency factors, and indirectly by shortening the G1 phase.

In several tumors, cyclin E overproduction has also been reported through *CCNE1* gene amplification, the transcriptional activation of *CCNE1* by c-MYC or E2F transcription factors, or the inactivation of the FBXW7 ubiquitin ligase adapter (reviewed in [52]). While cyclin E-CDK2 activity protects pluripotency in ESCs, it causes replication stress in the S phase by inducing incomplete DNA replication and abnormal mitotic progression in cancers [53,54].

Interestingly, cyclin D overproduction is implicated in multiple cancers, which leads to CDK4/6 hyperactivation and inactivation of the Rb tumor suppressor [55]. In contrast, cyclin D levels are remarkably low in mESCs and slightly higher in hESCs, though not to the same level as normal somatic cells, which renders CDK4/6 activity in mESCs minimal [11]. In hESCs, cyclin D1-D3 levels exhibit canonical cell-cycle-dependent oscillations: low in early G1 and peaks in late G1. When cyclin D-CDK 4/6 activity is low in early G1, the SMAD2/3 transcription factors can bind and activate endoderm genes if endoderm differentiation signals are present. However, when cyclin D-CDK4/6 activity rises in late G1, CDK4/6 phosphorylates SMAD2/3 and prevents their nuclear translocation, thus inhibiting endoderm differentiation and allowing for neuroectoderm differentiation if neuroectoderm differentiation signals are present. Interestingly, manipulating cyclin D-CDK4/6 activity by overexpressing or knocking down cyclin D, or inhibiting CDK4/6 activity, can alter the cell fate choice of hESCs [47]. 

It is worth pointing out that live cell imaging of single mESCs might reveal rapid oscillations in cyclin levels, and CDK and E2F activities, that might be obscured by bulk biochemical methods in asynchronous ESC populations. In contrast to mESCs, hESCs exhibit the canonical oscillatory nature of cyclins, CDK activity, and RB phosphorylation [56]. Notably, hESCs represent a slightly later “primed” pluripotent state compared to the early mESCs “naïve” pluripotent state, which could explain the reported cell cycle differences between them [11]. It would be interesting to compare how the oscillatory versus constitutive cell cycle regulators in hESCs versus mESCs, respectively, affect their self-renewal and differentiation capacities. 

### 3.2. CDK Inhibitors

The rapid G1/S transition because of the sustained high CDK2 activity in hESCs is reinforced via low p27 and p21 (CDK inhibitor) expression. These CDK protein inhibitors are down-regulated because SKP2, the substrate adapter that drives their ubiquitination and degradation, is itself degraded via the APC/C: a multi-subunit ubiquitin ligase complex. hESCs, as well as several cancers, are characterized by high levels of SKP2 due to high levels of an APC/C inhibitor (EMI1), which in turn limits p27 expression [57,58,59] (reviewed in [60]). Moreover, p21 transcription is suppressed in hESCs via H3K27 trimethylation of the p21 promoter, which is deposited by the EZH2 methyltransferase. Upon differentiation, the H3K27 trimethylation is rapidly lost and p21 expression is activated by p53 [61,62]. Interestingly, deletions of p21, p27, and p18 (another CDK inhibitor) enhance reprogramming efficiency [51]. 

In most cancers, mutations of *TP53* (the gene encoding the p53 tumor suppressor) or the loss of function of p53 are frequent, which result in low p21 levels because the transcription of the gene encoding p21 is dependent on p53 (reviewed in [43,63]). Taken together, p21 and p27 levels are low in hESCs and some cancers, which contributes to an abbreviated G1 phase, pluripotency maintenance, and tumor proliferation, respectively. The relative expression levels or activities of different cyclins, CDKs, CDK inhibitors, origin licensing factors, and some of the pluripotency factors in stem cells and cancer cells, relative to somatic cells, are summarized in Table 1.

## 4. G1 Origin Licensing Dynamics in Stems Cells and Cancer

Origin licensing involves the cooperative action of licensing factors to load MCMs onto DNA in G1 phase. Briefly, the heterohexameric origin recognition complex (ORC), made up of ORC1-6, binds to DNA. Cell division cycle 6 (CDC6) then binds to ORC, and the CDC10-dependent transcript 1 protein (CDT1) binds one MCM2-7 heterohexamer, which together associate with the DNA-bound ORC-CDC6, which load the first MCM2-7 hexamer onto DNA. Then, the second MCM2-7 hexamer, escorted by CDT1, is loaded by ORC-CDC6 onto DNA with the N-termini of the two MCM hexamers facing each other. A double MCM hexamer is stably loaded onto DNA in an inactive form until origin firing starts in S phase (reviewed in [64,65]). 

We have previously discovered that stem cells load the same amount of MCMs as their differentiated counterparts in much less time (due to a short G1), and therefore MCMs are loaded at a faster rate [10]. Some cancer cells might exhibit either inappropriate licensing or underlicensing. We will discuss below some similarities and differences between stem cells and cancer cells in their origin licensing dynamics.

### 4.1. Origin Licensing Factors

CDT1 is normally tightly regulated via several independent mechanisms to control its function outside of G1 phase (reviewed in [66]). Dysregulated CDT1 expression can result in the re-firing of the same origins in S phase, known as re-replication, as well as defects in chromosome segregation in mitosis [67,68], which compromises the genome integrity and predisposes for malignant transformation [69]. *Cdt1* transcription is controlled by the Rb-E2F pathway, which is frequently deregulated in cancers to drive the aberrant overexpression of E2F target genes [70]. Indeed, CDT1 and CDC6 overexpression is reported in the early stages of hyperplasia, which together with defects in p53-mediated senescence results in more aggressive tumors and chemoresistance [71,72]. Moreover, CDT1 and CDC6 overexpression leads to DNA re-replication and tissue dysplasia in mice [73]. Interestingly, ESCs and iPSCs express high levels of CDT1 in G1 phase only, which is degraded in S phase by the E3 ubiquitin ligases, CRL4^Cdt2^ and CRL1^Skp2^, to prevent re-replication [10,57,74].

CDC6 levels are high in cancer cells and ESCs due to high E2F-dependent transcription [57,70,71]. Notably, CDC6 is normally targeted for ubiquitin-mediated proteolysis by the APC/C in G1 phase [75]. EMI1, the APC/C inhibitor, is highly expressed in several cancers as well as in ESCs, but it significantly decreases upon differentiation. Thus, EMI1-mediated inhibition of APC/C activity in G1 phase also contributes to the sustained elevated levels of CDC6 [57,76,77]. High levels of CDC6 in cancers induce DNA over-replication, inhibit apoptosis, and increase tumor invasiveness and metastasis [67,78,79,80]. In ESCs, high levels of CDC6 might allow for rapid origin licensing to counteract the sustained high CDK activity in G1 phase that normally inhibits origin licensing [57]. 

Point mutations in MCM4 disrupt and destabilize the MCM2-7 complex function in S phase, which might lead to incomplete DNA replication in skin and endometrial cancer, respectively [81,82]. 

Taken together, some origin licensing factors are overexpressed or deregulated in stem cells and some cancers. However, manipulating the levels of the licensing factors is not sufficient to largely alter licensing rates. For example, overproducing CDT1 alone does not induce fast licensing in untransformed epithelial cells, so high levels of CDT1 cannot fully explain the fast licensing in iPSCs or some cancer cells. Moreover, expressing a mutant version of CDC6 that is resistant to APC/C degradation slightly increases origin licensing in untransformed epithelial cells but it does not induce a short G1, indicating that G1 length and licensing are controlled via distinct mechanisms [10]. The total levels of MCM subunits are also not rate-limiting for origin licensing since they are ubiquitously expressed in cells. It could be that cancer cells and stem cells employ other distinct pathways which, in addition to the overproduction of licensing factors, control origin licensing rates.

### 4.2. Dormant Origins in Cancer versus Stem Cells

As we discussed above, the increased expression of origin licensing factors in cancers and stem cells might allow these cells to license a high level of “dormant origins”. In a typical S phase, many origins that were licensed in the previous G1 are never utilized but are instead passively removed during DNA replication. The presence of these extra licensed origins becomes important at regions of DNA damage or stalled replication forks. The ability to fire local origins around stalled forks helps to ensure complete DNA replication. Since S phase cells cannot license new origins, the extra licensing to provide rescue origins must occur in G1. These back-up origins are known as “dormant origins”—not to be confused with dormant cells—and they are particularly essential when cells are challenged with high levels of replication stress in S phase [83,84,85]. Indeed, ESCs license more dormant origins, and partial depletion of the dormant origins via MCM knock-down does not affect their self-renewal, but impairs their neural differentiation potential [27]. Moreover, we have shown before that knocking down CDT1 or CDC6 in hESCs slowed down licensing and promoted differentiation [10]. Similarly, cancer cells are more sensitive to the depletion of origin licensing factors than untransformed cells [86]. This increased sensitivity of cancer cells might be explained by the excessive reliance on dormant origins to counteract high levels of replication stress, or underlicensing, which already limits the pool of available licensed origins [22].

Interestingly, while both stem cells and cancer cells exhibit excessive replication stress and origin firing, ESCs proficiently resolve most S phase-associated damage before entering mitosis by employing highly effective replication-coupled repair mechanisms. It is also plausible that ESCs can tolerate residual stress from S phase during mitosis to maintain rapid cell cycles before differentiation [87]. However, in cancers, excessive origin firing triggers chromosome mis-segregation in mitosis, and chromosomal instability that fails to be resolved [88]. 

Origin licensing must occur evenly across all genomic regions to ensure complete DNA replication. An uneven distribution of MCM complexes might result in under-replication if specific genomic regions are favored over other regions (reviewed in [89]). The genomic distribution of origin firing might also be different in stem cells versus cancer cells. Origin firing from dormant origins in cells experiencing replication stress initiates from novel unutilized origins that are evenly distributed, which might ensure complete replication of the genome in ESCs. Conversely, in cancers, Cdt1-mediated re-replication might occur unevenly, where early-replicating origins, but not late-replicating origins, preferentially re-fire again within the same S phase, which could lead to genome instability [90]. Notably, iPSCs generated from adult cells showed less origin licensing and firing, which led to a lower differentiation potential and increased genome instability, compared to ESCs generated via somatic cell nuclear transfer (SCNT) [91]. Thus, ESC-specific protective mechanisms might be lacking in iPSCs, which could explain the tumorigenic potential of iPSCs [92,93]. These discrepancies between ESCs, iPSCs, and cancer cells might prime novel mechanistic studies on adult stem cells that show aging-related functional attrition, as well as a propensity to undergo tumorigenesis [94,95].

## 5. Cell Cycle Regulation by the Reprogramming Factors in Stem Cells and Cancer

Somatic differentiated cells can be reprogrammed into induced pluripotent stem cells (iPSCs) by the four “Yamanaka reprogramming factors” (SOX2, OCT4, KLF4, and c-MYC), or different combinations of other transcription factors or chemical compounds [14,96,97]. Moreover, these factors—among others—comprise a core pluripotency network in embryonic stem cells (ESCs), but they are normally silenced in normal differentiated cells, except for some tissue-specific adult stem cells. Interestingly, some cancers can aberrantly re-express some of these factors, which contribute to a resistance to cancer therapy [98]. We will discuss below how SOX2, OCT4, KLF4, and c-MYC regulate G1 dynamics in stem cells and cancer cells.

### 5.1. SOX2

Sex-determining region Y-box 2 (SOX2) is a key transcription factor that blocks differentiation and maintains self-renewal in stem cells [99]. For example, SOX2 protects the limited pool of neural stem cells in the cortex by maintaining them in a slow-cycling state, which can shift into a proliferating state upon differentiation into intermediate progenitor cells. SOX2 represses *CCND1* (the gene encoding cyclin D1) in neural stem cells by binding to low-affinity motifs and facilitating the recruitment of other corepressors. As neural stem cells differentiate, pre-neural factors reduce SOX2 levels, which in turn derepresses *CCND1* and induces proliferation [100]. Moreover, SOX2 directly upregulates *CDKN1B* (the gene encoding the CDK inhibitor p27) to maintain the quiescence of the inner pillar cells in the postnatal sensory epithelium [101]. In normal gastric mucosae, SOX2 is normally expressed, but it is downregulated in gastric carcinomas. SOX2 overexpression in gastric epithelial cells leads to cell cycle arrest, decreased levels of cyclin D1 and phosphorylated Rb, and increased levels of p27. Thus, the loss of SOX2 in gastric carcinomas could contribute to tumorigenesis by inducing proliferation [102]. Moreover, several SOX2^high^ cancer stem cells are quiescent but they retain their tumor-initiating capacity (reviewed in [103]). 

On the other hand, SOX2 was found to directly or indirectly activate cyclins D1, A2, and B1 to induce proliferation in lung, head, and neck carcinoma [104,105,106]. Moreover, SOX2 directly represses *p21* to drive G1 progression in lung cancer [107]. Thus, the role of SOX2 in cell cycle regulation in different cancer contexts might be highly dose-dependent [5,108]. Indeed, SOX2 levels must be tightly controlled; small changes either through depletion or overexpression impair ESCs’ self-renewal, trigger their differentiation, and inhibit tumor growth [109,110]. This could be explained by the highly complex and interconnected network in which SOX2 functions in stem and cancer cells [111,112]. 

### 5.2. OCT4

OCTamer-binding transcription factor 4 (OCT4) is one of the key components of the core pluripotency network [113]. OCT4 plays a pivotal role in maintaining the pluripotency and self-renewal of ESCs, generating iPSCs as well as driving cancer cell proliferation (reviewed in [114,115]).

In several cancers and in adult stem cells, OCT4 drives cells through the G1/S transition by directly binding to the *CCND1* gene promoter as well as inducing cyclin E expression [116,117,118,119,120]. OCT4 also directly induces *E2F3* in mESCs, and indirectly represses p21 through the upregulation of DNMTs, which methylates and silences the *CDKN1A* gene promoter, which encodes p21, in mesenchymal stem cells [121,122,123]. Moreover, OCT4 induces the expression of protein phosphatase 1 (PP1) inhibitors: the nuclear inhibitor of PP1 (NIPP1) and cyclin F (CCNF), which prevents the PP1-dependent de-phosphorylation of Rb, and accelerates cell proliferation in ovarian cancer [124]. This OCT4-NIPP1/CCNF-PP1-pRb axis is important for maintaining self-renewal in mESCs, too [124,125]. Interestingly, OCT4 safeguards the genome integrity of the rapidly proliferating ESCs by forming a complex with cyclin-CDK1, which blocks CDK1 activation and premature entry into mitosis [126]. Whether similar protective mechanisms exist in tumor initiating cells with detectable levels of OCT4 is not yet investigated [6]. 

### 5.3. KLF4

Krüppel-like factor 4 (KLF4) was initially identified as an inhibitor of cellular proliferation where its levels are low in actively dividing cells, but they remarkably increase in serum-starved or contact-inhibited cells [127]. KLF4 blocks the G1/S transition and inhibits cell cycle progression after DNA damage by recruiting p53 to the *CDKN1A* promoter, and thereby inducing *CDKN1A* (the gene encoding the CDK inhibitor p21) in colon cancer [128,129]. KLF4 also inhibits the tumor proliferation and metastasis of non-small cell lung cancer (NSCLC) through downregulating cyclin D1, upregulating p21, and inhibiting MSI2: an activator of the JAK/STAT3 signaling pathway that promotes metastasis [130]. 

However, KLF4 can bypass RAS(V12)-mediated senescence and confer resistance to DNA-damage-induced apoptosis in mouse embryonic fibroblasts by directly binding to the p53 promoter and suppressing its expression [131]. Moreover, the KLF4/MUC5AC axis—the most abundant mucin in pancreatic cancer—is suggested to maintain self-renewal in the early stages of pancreatic ductal adenocarcinoma (PDA) [132]. However, KLF4 inhibits proliferation in advanced PDA stages through p27 induction at the RNA and protein levels, along with other factors [133]. Thus, the role of KLF4 as an oncogene or tumor suppressor is dependent on the status of other proteins such as p21^CIP1^, the cancer type, and the developmental stage [131,134]. In the context of reprogramming into iPSCs, the role of KLF4 might be the “brake”, to maintain genome integrity, while the role of other factors, such as c-MYC, might be the “accelerator”, to promote cellular proliferation [135,136].

### 5.4. c-MYC

c-MYC is a major stimulator of cell cycle progression and a repressor of differentiation genes during reprogramming. Since somatic cells undergoing reprogramming need to cycle, turn off the differentiation transcription program, and turn on the pluripotency program, c-MYC is thought to be needed for pluripotency establishment [137]. However, the mechanistic contribution of c-MYC to the sequential reprogramming phases and its interaction with the other pluripotency factors is still open to question [138]. Importantly, c-MYC can transform cultured cells when overexpressed, and its deregulation—through various mechanisms—is implicated in 70–80% of hematopoietic and solid tumors [139]. Thus, c-MYC plays a pivotal dual role in promoting iPSCs’ generation as well as tumor transformation in numerous malignancies [7].

c-MYC is also capable of forcing quiescent cells to re-enter the cell cycle in the absence of any mitogen stimulation [140,141]. The direct or indirect induction of cyclins, CDKs, and E2F activators, as well as the suppression of CDK inhibitors, are the main mechanisms through which c-MYC drives cells through the G1/S transition. In addition, c-MYC was reported to induce several of the origin licensing genes: *CDC6*, *CDT1*, and several of the *ORC* and *MCM* genes (reviewed in [142,143]). c-MYC also directly binds to some of the origin licensing factors and stimulates aberrant origin firing in G1 and S phases, respectively [19,144]. However, the physical association of c-MYC with factors involved in DNA replication is not sufficient to prove causality [145].

Does c-MYC regulate DNA replication through only its transcriptional role? We have recently shown that global hyperacetylation facilitates origin licensing on heterochromatin [146]. Moreover, it has been shown that histone deacetylase (HDAC) inhibitors such as valproic acid, together with OCT4, KLF4, and SOX2, can substitute for c-MYC, and efficiently generate iPSCs that resemble ESCs [147]. Does c-MYC facilitate origin licensing and firing by inducing a global open chromatin state [148]? Addressing these outstanding questions will help to identify the downstream effectors of c-MYC that maintain pluripotency in iPSCs as well as oncogene addiction in MYC-driven tumors.

## 6. Conclusions and Future Directions

**Figure 3:**
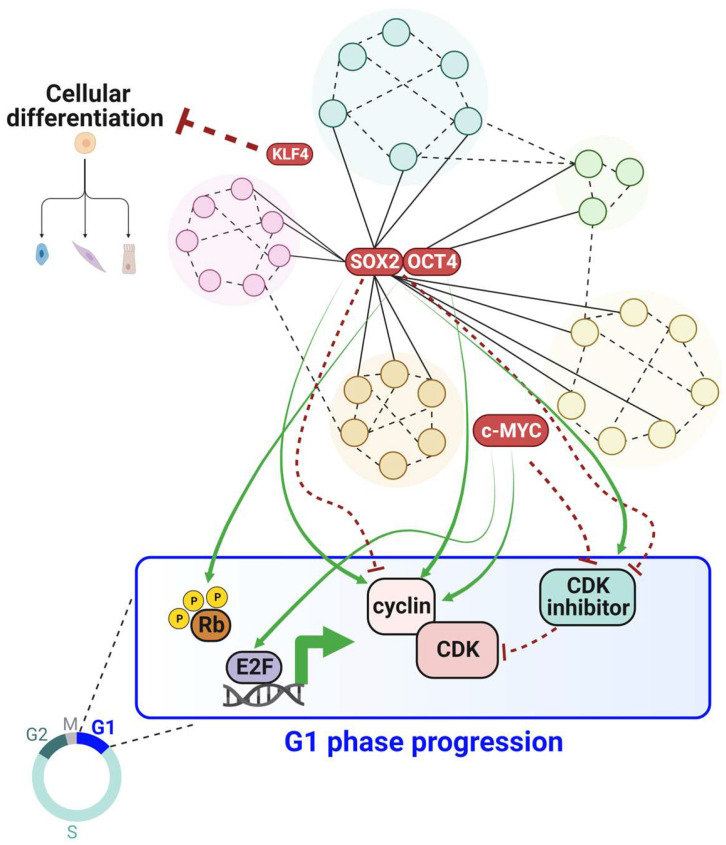
Illustration of the link between the pluripotency network and G1 phase progression. SOX2 and OCT4 govern a complex core pluripotency proteome network, which together with other factors such as NANOG (not depicted here), KLF4, and c-MYC, shortens G1 to block differentiation and maintain pluripotency. SOX2 has dose-specific and context-dependent functions that are depicted as green arrows for activation or red dashed arrows for inhibition. Created with BioRender.com, accessed on 29 August 2023.

Successful iPSC reprogramming is driven by the pluripotency factors in a complex regulatory network that is highly dependent on their stoichiometry and timing of expression [149,150]. Interestingly, cells that successfully reprogram acquire a uniquely rapid proliferation rate in the initiation phase of reprogramming [151]. Moreover, overexpressing cyclin D1 or knocking down p53 increases the number of cells amenable for reprogramming into iPSCs and promotes the maturation phase of reprogramming [152]. We also highlighted the context-dependent functions of some of the pluripotency factors in driving G1 progression (Figure 3). Which of the four pluripotency factors have the biggest effect on accelerating the proliferation rate and inducing the short G1 characteristic of stem cells? Notably, changes in the levels of cyclins, CDKs, or CDK inhibitors after manipulating the levels of pluripotency factors might only be a symptom of cell cycle changes in asynchronous populations, rather than a direct cause. Thus, identifying the molecular mechanisms by which pluripotency factors control cell cycle dynamics might need careful investigation in each cell cycle phase on a single-cell level. Moreover, little is known about the role of these pluripotency factors in regulating origin licensing. Do these factors accelerate origin licensing rates to counterbalance the short G1 phase and preserve genome integrity in S phase? We also discussed that these pluripotency factors are aberrantly expressed in some cancers (Table 1); however, the exact molecular mechanisms by which they regulate the cell cycle dynamics in different tumor contexts warrant further investigation. Answering these questions will help to identify common developmental pathways between tumorigenesis and stem cell pluripotency.

## Figures and Tables

**Table 1:** Relative expression and activity of cell cycle regulators and pluripotency factors.

Cell Cycle Regulator	Stem Cells versus Somatic Cells	Some Cancers versus Somatic Cells	Section
**Cyclins**			Section 3.1
**Cyclin E**	Higher levels	Higher levels	
**Cyclin D**	Intermediate levels	Higher levels	
**Cyclin A**	Higher levels	Higher levels	
**Cyclin-dependent kinases (CDKs)**			
**CDK2**	Higher activity	Higher activity	
**CDK4/6**	Intermediate activity	Higher activity	
**CDK1**	Higher activity	Higher activity	
**CDK inhibitor proteins**			Section 3.2
**p27**	Lower levels	Lower levels	
**p21**	Lower levels	Lower levels	
**Origin licensing factors**			Section 4.1
**CDC6**	Higher levels	Higher levels	
**CDT1**	Higher levels	Higher levels	
**MCM loading**	Faster loading in G1	? *	
**Pluripotency Factor**	**Stem Cells versus Somatic Cells**	**Some Cancers versus Somatic Cells**	Section 5
**SOX2**	Higher levels	Higher levels in a subset of cancer cells	Section 5.1
**OCT4**	Higher levels	Higher levels in a subset of cancer cells	Section 5.2
**KLF4**	Higher levels	Expression levels are cancer-specific	Section 5.3
c-MYC **	?	Higher levels in most cancers	Section 5.4

* MCM loading rates have not been directly measured in different cancer types. ** c-MYC is frequently used to reprogram to pluripotency, but is not a pluripotency facter per se. The levels of c-MYC in embryonic stem cells (ESCs) and differentiated somatic cells have not been directly compared or quantified.
